# Assessment of Prevalence and Risk Factors of Diabetes Distress Among Patients With Type 2 Diabetes Mellitus in an Urban Area of Chengalpattu District, India: A Cross-Sectional Study

**DOI:** 10.7759/cureus.98974

**Published:** 2025-12-11

**Authors:** Nikhil C M, Shanthi Edward, Angeline Grace, Swetha N B

**Affiliations:** 1 Community Medicine, Sree Balaji Medical College and Hospital, Chennai, IND

**Keywords:** anxiety, depression, diabetes distress, diabetes mellitus, psychological distress

## Abstract

Background and aim

Type 2 diabetes mellitus (T2DM) is a chronic metabolic disorder associated with significant physical and psychological complications, including diabetes distress, which is the emotional burden of living with and managing diabetes, adversely affecting glycemic control. It includes emotional, regimen-related, interpersonal, and physician-related distress. The global prevalence varies widely, while Indian data remain limited and inconsistent. Factors such as fear of complications, treatment fatigue, inadequate support, and poor doctor-patient communication contribute to this condition, which often goes unrecognized. This study aims to estimate the prevalence of diabetes distress and determine its risk factors among T2DM patients residing in an urban area of the Chengalpattu district.

Methodology

A cross-sectional study was conducted among T2DM patients in Anakaputhur, an urban area of the Chengalpattu district, Tamil Nadu, India. A total of 297 people participated in the study. A pretested, structured questionnaire was used to collect socio-demographic details, diabetes history, complication history, economic history, and activities of self-care. The validated Type 2 Diabetes Distress Assessment System (T2DDAS) was used to screen for diabetes distress. Data analysis was performed using IBM SPSS Statistics for Windows, Version 25 (Released 2017; IBM Corp., Armonk, NY, USA).

Results

The prevalence of diabetes distress among T2DM patients was 35.6%. Being on treatment with both oral hypoglycemic agents (OHA) and insulin, having a diabetic foot ulcer, and not having adequate physical activity were found to be significantly associated with diabetes distress. The history of diabetic retinopathy and cardiac comorbidities was also found to be significantly associated with diabetes distress.

Conclusion

It was found that 35.6% of patients with T2DM suffered from diabetes distress, indicating a significant, but mostly unrecognized, health problem. These findings suggest that the interrelated consequences of clinical, psychological, and social issues in diabetes management warrant routine screening as part of diabetes care services. Distress management should be incorporated into the National Program for Non-communicable Diseases (NP-NCD) to enable early identification and support, helping to improve treatment adherence and quality of life by strengthening community healthcare.

## Introduction

Type 2 diabetes mellitus (T2DM) is a chronic medical condition characterized by persistently elevated blood sugar levels, occurring when the pancreas either cannot produce enough insulin or the body cannot utilize insulin effectively. Uncontrolled T2DM, which usually results from improper management, can give rise to complications affecting the heart (cardiovascular diseases), eyes (diabetic retinopathy), kidneys (diabetic nephropathy), nerves (diabetic neuropathy), non-healing diabetic foot ulcers, hearing loss, and sexual dysfunction [1]. Beyond the physical aspects of diabetes management lies a significant psychological dimension that has gained increasing recognition in recent decades. The emotional response of a person to diabetes mellitus - from living with the disease, managing it on a regular basis, and dealing with its complications - is called “diabetes distress.” [2].

Diabetes distress is defined across four broad themes, namely emotional distress, regimen distress, interpersonal distress, and physician distress. Emotional distress occurs from the onset of the disease and is accompanied by anger, frustration, and fear of complications of T2DM. When individuals feel overwhelmed by diabetic medications and become non-compliant, regimen distress occurs. Lack of social support can lead to interpersonal distress. Physician distress arises when there are lacunae in the doctor-patient relationship and in the transfer of knowledge between the person with diabetes and the treating physician [3].

Globally, the burden of diabetes distress varies across countries, with 1.4% in Germany, around 4% in the Netherlands, about 8.9% in Thailand, 9.3% to 21% in the United States, 22.3% in Saudi Arabia, and about 24.4% in Greece [4-10]. In India, studies on diabetes distress are limited. A study conducted in Chennai found the prevalence to be 61%, a study conducted in urban and rural areas of Punjab found that 18% had diabetes distress, and a study in Delhi found the prevalence to be 35.4% [11-13].

Many factors may be responsible for the causation of diabetes distress, the most important being feelings of being overwhelmed, angry, frustrated, and burned out due to living with a chronic disease. Social factors may include a lack of empathy from family members in understanding the demands of this condition, an inability to maintain food restrictions, and fear of developing complications [14]. This emotional distress, which is mostly hidden from the healthcare provider, is found to be associated with underdiagnosis and worsening glycemic control despite the use of medications, ultimately leading to a poor quality of life in these individuals with diabetes [15].

Though diabetes distress can be treated adequately, if it is not addressed, it can worsen glycemic control, leading to chronic complications [16]. Psychosocial care is widely recommended to complement the regular treatment of T2DM. However, this seldom happens, and diabetes distress remains an undiagnosed and underdiagnosed medical condition in countries like India, where stigma regarding mental health is widely prevalent [17].

Current healthcare systems recognize that in many diseases, along with physiological treatment, focusing on psychosocial components yields better outcomes for various chronic conditions. While the physical complications of diabetes have been extensively studied, the emotional and psychological dimensions of living with the disease have been neglected in the treatment of diabetes at large. There is also a dearth of studies on diabetes distress in India. The scarcity of diabetes distress research in India, combined with widespread mental health stigma in low- and middle-income settings, underscores the urgent need for comprehensive studies to understand the burden and contributing factors of this condition, thereby informing effective management and prevention strategies. Based on the above background, the study was carried out in an urban area of the Chengalpattu district with the following objectives: (1) to estimate the prevalence of diabetes distress among T2DM patients residing in an urban area of the Chengalpattu district, and (2) to determine the risk factors associated with diabetes distress among T2DM patients residing in an urban area of the Chengalpattu district.

## Materials and methods

Study design and setting

A community-based cross-sectional study was conducted among T2DM patients in Anakaputhur, an urban area of the Chengalpattu district, Tamil Nadu, India. A total of 297 people participated in the study from July 2023 to December 2024 (18 months).

Inclusion and exclusion criteria

The inclusion criteria were known diabetics for a minimum period of one year, and T2DM patients with comorbidities such as hypertension, hyperlipidemia, coronary heart disease, and stroke. The exclusion criteria were T2DM patients who were bedridden, unable to communicate, or had a known mental illness.

Sample size and calculation

A study conducted by Patra et al. [18] in North India reported a prevalence of diabetes distress of 42%. This prevalence was used to calculate the sample size using the Dobson formula, $n = \frac{Z^2 \cdot p \cdot (1-p)}{L^2}$. Assuming a non-response rate of 10%, the sample size was revised from 260 to 260 + 10% (26) = 286, which was then rounded off to 290.

Sampling method

The total adult population of Anakaputhur was 30,807, who were distributed among four wards with populations of Ward 1 - 7,180; Ward 2 - 7,818; Ward 3 - 7,854; and Ward 4 - 7,955. Based on a study by Pradeepa and Mohan et al., T2DM prevalence in urban Tamil Nadu is 13.7% [19]. Estimating this value for our field practice area, the total adult population with T2DM is approximately 4,250. Distributing this diabetic population among the four wards, the estimated diabetic population in each ward was calculated to be approximately 1,100. The sample size required for our study was 290, which was proportionately distributed among the four wards using probability-proportional-to-size sampling (PPS sampling). Applying PPS sampling, the required sample size was 68 for Ward 1, 73 for Ward 2, 74 for Ward 3, and 75 for Ward 4. To achieve this, a door-to-door survey was conducted in each ward, starting from the first house of the street at the center of the ward until the required sample size was achieved. If two or more diabetic patients were present in a house, the study participant was chosen randomly. If the person was not willing to participate in the study or if no one was present in the house, the next house in line was approached.

Study tools

A pretested, structured questionnaire, including socio-demographic details, diabetes history, complication history, comorbidity history, economic history, and activities of self-care (physical activity, dietary pattern, and diabetes monitoring), was used (see Appendix). The validated Type 2 Diabetes Distress Assessment System (T2DDAS) [20] was used to screen for diabetes distress among the T2DM patients. This tool is a 29-item questionnaire that collects details about many stresses and worries that people with type 2 diabetes often experience. Each item is scored on a five-point Likert scale ranging from 1 to 5, with 1 meaning ‘Not a problem’, 2 meaning ‘A little problem’, 3 indicating ‘A moderate problem’, 4 being ‘A serious problem’, and 5 being ‘A very serious problem’. Responses to each item are summed, and the average score is calculated to screen for the presence of diabetes distress in the participant. Based on the mean score, the distress levels of the participants are classified as: <2.0 - little or no distress, 2.0-2.9 - moderate distress, and ≥3.0 - high distress.

Data analysis

Data were entered into Microsoft Excel (Microsoft® Corp., Redmond, WA, USA), and statistical analysis was performed using IBM SPSS Statistics for Windows, Version 25 (Released 2017; IBM Corp., Armonk, NY, USA). Descriptive statistics are presented in tables and graphs. Bivariate analysis was performed using the Chi-square test, and variables that were found to be statistically significant at the 95% confidence interval (CI) were included in the logistic regression model.

Ethics approval and informed consent

Ethics approval was obtained from the Institutional Human Ethics Committee, Sree Balaji Medical College and Hospital, Chennai, India, with approval no. 002/SBMCH/IHEC/2023/2031. Informed consent was obtained from the study participants. The information was collected from the participants using a pretested, structured, and validated questionnaire, which was administered and documented by the researcher.

## Results

The sociodemographic details of the participants are given in Table 1. A total of 297 individuals with T2DM participated in the study. Of these, 157 (52.9%) participants were above 56 years of age, and 159 (53.5%) were males. Socioeconomic status was assessed using the BG Prasad classification, and it was found that 168 (56.6%) participants belonged to the upper class.

**Table 1:** Sociodemographic details of the study participants

S. No.	Variable	Frequency (n)	Percentage (%)
1	Age		
	More than 56 years	157	52.9
	Less than or equal to 55 years	140	47.1
2	Gender		
	Female	138	46.5
	Male	159	53.5
3	Educational Qualification		
	Illiterate	5	1.7
	Primary School	46	15.5
	Middle School	113	38
	High School	89	30
	Graduate/Diploma	35	11.8
	Postgraduate/Professions of Honour	9	3
4	Occupation		
	Unemployed	13	4.4
	Unskilled Worker	1	0.3
	Semi-skilled Worker	207	69.7
	Skilled Worker	56	18.9
	Professionals	20	6.7
5	Marital Status		
	Married	267	89.9
	Never Married	3	1
	Separated	2	0.7
	Widowed	25	8.4
6	Socioeconomic Status (BG Prasad Classification)		
	Lower Class	30	10.1
	Lower Middle Class	17	5.7
	Middle Class	26	8.8
	Upper Middle Class	56	18.9
	Upper Class	168	56.6
7	Religion		
	Hindu	250	84.2
	Muslim	20	6.7
	Christian	27	9.1

Factors related to diabetes distress among the study participants are given in Table 2. In terms of duration of diabetes, most participants (202, 68%) had been diagnosed for more than five years. Regarding treatment, 61 (20.5%) were on combination therapy (both oral hypoglycemic agents (OHA) and insulin), while the majority (236, 79.5%) were receiving either OHA or insulin alone. Physical activity levels were reported as adequate by 236 (79.5%) participants. In terms of dietary compliance, 225 (75.8%) participants followed a diabetic diet. Regular glucose monitoring and follow-up practices were reported by 222 (74.7%) participants.

**Table 2:** Factors related to diabetes distress among the study participants OHA, Oral Hypoglycemic Agents

S. No.	Variable	Frequency (n)	Percentage (%)
1	Duration of Diabetes		
	More Than or Equal to 5 Years	202	68
	Less Than 5 Years	95	32
2	Diabetes Treatment		
	OHA+Insulin	61	20.5
	OHA/Insulin	236	79.5
3	Physical Activity		
	Inadequate	61	20.5
	Adequate	236	79.5
4	Diabetic Diet		
	Doesn’t Follow	72	24.2
	Follows Diabetic Diet	225	75.8
5	Regular Glucose Monitoring and Follow-Up		
	No	75	25.3
	Yes	222	74.7

Details about the comorbidity history are given in Table 3. Peripheral neuropathy was the most prevalent comorbidity, reported by 254 (85.5%) participants. A history of diabetic foot ulcers was present in 135 (45.5%) participants. Diabetic retinopathy was present in 180 (60.6%) participants. Diabetic nephropathy was reported by 231 (77.8%) participants. Hypertension was identified in 139 (46.8%) participants. Cardiac comorbidities, such as ischemic heart disease or congestive heart failure, were present in 135 (45.5%) participants. A history of stroke was reported by 109 (36.7%) participants.

**Table 3:** Comorbidities in study participants

S. No.	Variable	Frequency (n)	Percentage (%)
1	H/O Peripheral Neuropathy		
	Yes	254	85.5
	No	43	14.5
2	H/O Diabetic Foot Ulcer		
	Yes	135	45.5
	No	162	54.5
3	H/O Diabetic Retinopathy		
	Yes	180	60.6
	No	117	39.4
4	H/O Diabetic Nephropathy		
	Yes	231	77.8
	No	66	22.2
5	Hypertension		
	Yes	139	46.8
	No	158	53.2
6	Cardiac Comorbidities		
	Yes	135	45.5
	No	162	54.5
7	H/O Stroke		
	Yes	109	36.7
	No	188	63.3

In this study, 191 (64.3%) participants had little or no distress, 77 (25.9%) had moderate distress, and 29 (9.7%) had high distress. For this study, people with moderate and high distress were considered to have diabetes distress. The bivariate analysis of sociodemographic factors and diabetes distress is shown in Table 4. Among the sociodemographic variables examined for association with diabetes distress, gender, occupation, and marital status showed statistically significant associations. Diabetes distress was significantly more prevalent among females (n = 58, 42%) (p = 0.034, OR = 1.677; 95% CI: 1.03-2.70). Participants who were unemployed, unskilled, or semi-skilled had higher distress levels (n = 86, 38.9%) compared to skilled workers and professionals (n = 20, 26.3%) (p = 0.048, OR = 1.78; 95% CI: 1.01-3.17). Unmarried, separated, or widowed individuals had a higher prevalence of diabetes distress (n = 16, 53.3%) compared to married individuals (n = 90, 33.7%) (p = 0.033, OR = 2.24; 95% CI: 1.05-4.81). Other variables did not show statistically significant associations with diabetes distress.

**Table 4:** Association between sociodemographic details of study participants and diabetes distress *p-value < 0.05, statistically significant at the 95% confidence interval.

S. No.	Variable	Diabetes Distress		Chi-Square		p-value	Odds Ratio	OR 95% CI
		Yes, n (%)	No, n (%)					
1	Age							
	More Than 56 Years	57 (36.3)	100 (63.7)	0.055		0.815	1.059	0.65-1.70
	Less Than or Equal to 55 Years	49 (35)	91 (65)					
2	Gender							
	Female	58 (42)	80 (58)	4.513		0.034*	1.677	1.03-2.70
	Male	48 (30.2)	111 (69.8)					
3	Educational Qualification							
	Illiterate/Primary School/Middle School/High School	95 (37.5)	158 (62.5)	2.572		0.109	1.804	0.87-3.73
	Graduate/Diploma/Postgraduate/Professions of Honour	11 (25)	33 (75)					
4	Occupation							
	Unemployed/Unskilled/Semiskilled Workers	86 (38.9)	135 (61.1)	3.91		0.048*	1.78	1.01-3.17
	Skilled/Professionals	20 (26.3)	56 (73.7)					
5	Marital Status							
	Unmarried/Divorced/Widowed	16 (53.3)	14 (46.7)	4.526		0.033*	2.24	1.05-4.81
	Married	90 (33.7)	177 (66.3)					
6	Socio-Economic Status							
	Lower Class/Lower Middle Class/Middle Class/Upper Middle Class	50 (38.8)	79 (61.2)	0.936		0.333	1.266	0.78-2.04
	Upper Class	56 (33.3)	112 (66.7)					
7	Religion							
	Hindu	89 (35.6)	161 (64.4)		0.006	0.94	0.976	0.51-1.86
	Muslim/Christians	17 (36.2)	30 (63.8)					

Table 5 gives the bivariate analysis of diabetes distress, self-care activities, and other variables. Individuals on both OHA and insulin were more likely to report distress (n = 31, 50.8%) compared to those on either OHA or insulin alone (n = 75, 31.8%) (p = 0.006, OR = 2.218; 95% CI: 1.25-3.93). Participants with inadequate physical activity had higher distress (n = 29, 47.5%) (p = 0.03, OR = 1.871; 95% CI: 1.05-3.31). Those not adhering to a diabetic diet had greater distress (n = 33, 45.8%) compared to adherents (n = 73, 32.4%) (p = 0.039, OR = 1.762; 95% CI: 1.02-3.02). Participants not following regular monitoring had higher distress (n = 35, 46.7%) (p = 0.022, OR = 1.861; 95% CI: 1.09-3.17).

**Table 5:** Association between self-care practices and other factors with diabetes distress *p-value < 0.05, statistically significant at the 95% confidence interval. OHA, Oral Hypoglycemic Agents

S. No.	Variable	Diabetes Distress			Chi-Square	p-value	Odds Ratio	Odds Ratio 95% CI
		Yes, n (%)		No, n (%)				
1	Duration of Diabetes							
	More Than or Equal to 5 Years	77 (38.1)		125 (61.9)	1.623	0.203	1.402	0.83-2.36
	Less Than 5 Years	29 (30.5)		66 (69.5)				
2	Treatment							
	OHA+Insulin	31 (50.8)		30 (49.2)	7.656	0.006*	2.218	1.25-3.93
	OHA/Insulin	75 (31.8)		161 (68.2)				
3	Physical Activity							
	Inadequate	29 (47.5)		32 (52.5)	4.697	0.03*	1.871	1.05-3.31
	Adequate	77 (32.6)		159 (67.4)				
4	Diabetic Diet							
	Doesn’t Follow		33 (45.8)	39 (54.2)	4.260	0.039*	1.762	1.02-3.02
	Follows Diabetic Diet		73 (32.4)	152 (67.6)				
5	Regular Glucose Monitoring and Follow-Up							
	No		35 (46.7)	40 (53.3)	5.267	0.022*	1.861	1.09-3.17
	Yes		71 (32)	151 (68)				

Table 6 shows the bivariate analysis of comorbidities and diabetes distress. Those with peripheral neuropathy reported more distress (n = 97, 38.2%) than those without (n = 9, 20.9%) (p = 0.029, OR = 2.334; 95% CI: 1.07-5.07). Diabetic foot ulcer was associated with higher distress (n = 60, 44.4%) compared to those without (n = 46, 28.4%) (p = 0.004, OR = 2.017; 95% CI: 1.24-3.26). Participants with diabetic retinopathy had more distress (n = 75, 41.7%) than those without (n = 31, 26.5%) (p = 0.008, OR = 1.982; 95% CI: 1.19-3.28). Cardiac comorbidity was also significantly associated with distress (n = 60, 44.4% vs. n = 46, 28.4%) (p = 0.004, OR = 2.017; 95% CI: 1.24-3.26).

**Table 6:** Association between comorbidities and diabetes distress *p-value < 0.05, statistically significant at the 95% confidence interval.

S. No.	Variable	Diabetes Distress		Chi-Square	p-value	Odds Ratio	OR 95% CI
		Yes, n (%)	No, n (%)				
1	H/O Peripheral Neuropathy						
	Yes	97 (38.2)	157 (61.8)	4.772	0.029*	2.334	1.07-5.07
	No	9 (20.9)	34 (79.1)				
2	H/O Diabetic Foot Ulcer						
	Yes	60 (44.4)	75 (55.6)	8.264	0.004*	2.017	1.24-3.26
	No	46 (28.4)	116 (71.6)				
3	H/O Diabetic Retinopathy						
	Yes	75 (41.7)	105 (58.3)	7.111	0.008*	1.982	1.19-3.28
	No	31 (26.5)	86 (73.5)				
4	H/O Diabetic Nephropathy						
	Yes	88 (38.1)	143 (61.9)	2.62	0.106	1.641	0.89-3.00
	No	18 (27.3)	48 (72.7)				
5	Hypertension						
	Yes	54 (38.8)	85 (61.2)	1.136	0.287	1.295	0.80-2.08
	No	52 (32.9)	106 (67.1)				
6	Cardiac Comorbidity						
	Yes	60 (44.4)	75 (55.6)	8.264	0.004*	2.017	1.24-3.26
	No	46 (28.4)	116 (71.6)				
7	H/O Stroke						
	Yes	45 (41.3)	64 (58.7)	2.348	0.125	1.464	0.89-2.38
	No	61 (32.4)	127 (67.6)				

The results of multivariate logistic regression are shown in Table 7, and several independent predictors of diabetes distress were identified. Being employed in an unskilled, semi-skilled, or unemployed category significantly increased the odds (AOR = 2.694; p = 0.005). Patients who took both OHA and insulin therapy had a higher risk of distress (AOR = 3.190; p = 0.001). A history of diabetic foot ulcer (AOR = 3.774; p = 0.004), diabetic retinopathy (AOR = 1.777; p = 0.028), and cardiac comorbidities (AOR = 1.745; p = 0.014) were independently associated with higher diabetes distress. Inadequate physical activity (AOR = 2.834; p = 0.007) was also a strong predictor of diabetes distress.

**Table 7:** Multivariate logistic regression analysis of diabetes distress with related variables *p-value < 0.05, statistically significant at the 95% confidence interval.

S. No.	Variable	Diabetes Distress		
		AOR	95% CI	p-value
1.	Gender: Female	1.588	0.91-2.76	0.102
2.	Occupation: Unemployed/Unskilled/Semiskilled Workers	2.694	1.34-5.40	0.005*
3.	Marital Status: Unmarried/Divorced/Widowed	1.497	0.63-3.54	0.359
4.	Treatment: OHA + Insulin	3.190	1.59-6.38	0.001*
5.	H/O Peripheral Neuropathy: Yes	1.618	0.64-4.03	0.302
6.	H/O Diabetic Foot Ulcer: Yes	3.774	1.89-5.51	0.004*
7.	H/O Diabetic Retinopathy: Yes	1.777	1.11-3.25	0.028*
8.	H/O Cardiac Comorbidity: Yes	1.745	1.28-3.97	0.014*
9.	Physical Activity: Inadequate	2.834	1.32-4.17	0.007*
10.	Diabetic Diet: Doesn’t Follow	0.975	0.41-2.29	0.953
11.	Regular Glucose Monitoring and Follow-Up: No	1.471	0.58-3.72	0.415

## Discussion

The T2DM condition inherently causes distress in individuals due to their physical symptoms. This, coupled with psychological features like worry, frustration, and discouragement, gives rise to diabetic distress, which could lead to various symptoms that may affect the patient’s morbidity and mortality. The results observed among T2DM patients residing in an urban area of Anakaputhur have yielded interesting findings, which are discussed below.

In the current study, the prevalence of diabetes distress was 35.6%. A study conducted in Delhi by Alwani et al. yielded similar findings, with a frequency of 35.7% [13]. The prevalence was 42% in Odisha and 41.9% in Mangalore, according to studies conducted by Patra et al. [18,21]. According to a study conducted by AlOtaibi et al. in Saudi Arabia, 35.6% of people worldwide suffer from moderate to severe diabetes distress [22]. Surprisingly, a study conducted in rural areas of Punjab by Gupta et al. revealed that 100% of participants had diabetes distress, with 56% experiencing severe discomfort and 44% experiencing moderate distress [23]. This was attributed to the fact that access to diabetic medications and diabetic care was minimal in these rural areas. This alarmingly high prevalence of diabetes distress in T2DM patients is a cause for concern, as, if left untreated or unidentified, it could lead to problems related to medication adherence and disease outcomes. 

According to the study, people who were unemployed had a higher chance of receiving a diabetes distress diagnosis; approximately 39% of individuals who were unemployed or worked in unskilled labor had diabetes distress. Alwani et al.'s study produced similar findings [13]. Friis et al. discovered that, among diabetes patients, unemployment was one of the most significant indicators of distress [24]. Similar findings were reported by Symon et al., who found that diabetes distress was considerably higher among individuals who were unemployed or engaged in unskilled or semi-skilled labor [25]. This may be due to the fact that being unemployed or involved in unskilled/semiskilled work may add financial burden and physical exhaustion to the already morbid condition of T2DM, leading to greater distress in the individual. 

It was found that those who were on treatment with both OHA and insulin had increased odds of being diagnosed with diabetes distress compared to those who were on OHA alone, with around 50% of individuals on both OHA and insulin suffering from diabetes distress. Similarly, a study conducted by AlOtaibi et al. found that those on insulin had a statistically significant association with diabetes distress [22]. This may be due to fear of disease severity, injection-related anxieties, fear of hypoglycemia, and lifestyle disruption, as all activities, such as meal planning and exercise, must be coordinated with insulin use [26]. It should be understood by healthcare providers that starting a patient on an insulin regimen is not only a shift in their treatment but also a psychological transition, which must be well explained to the patient along with support systems to help them overcome stress related to insulin use.

The study found that individuals with a history of diabetic foot ulcers had a statistically significant association with diabetes distress. In a study conducted by Ullas et al. among patients with diabetic foot ulcers, it was found that around half of them were suffering from diabetes distress and had a poorer quality of life than those without ulcers [27]. The causes for this may be multifaceted. Firstly, individuals with diabetic foot ulcers live with the worry of amputation and losing their limbs. This could lead to poor self-care practices and non-adherence to a diabetic diet [28].

The present study found a statistically significant association between diabetic distress and diabetic retinopathy, with around 40% of individuals with a history of diabetic retinopathy experiencing diabetic distress. Similarly, a study conducted by Fenwick et al. found that diabetic retinopathy could lead to increased strain on family functioning, poor diabetes control, and accelerated progression of the disease, thereby leading to distress in the individuals [29]. It is imperative that patients are treated by specialists dealing with the retina so that problems can be identified at an early stage, and they can receive the necessary help and care. Similar recommendations were made in a study conducted by Bloom et al., which explored the role of retina specialists in alleviating fear and anxiety among T2DM patients with diabetic retinopathy, with emphasis on the primary and secondary prevention of the condition [30].

The presence of cardiac comorbidity along with T2DM, in the form of conditions such as myocardial infarction, angina, or cardiomyopathy, compounds the efforts an individual must make to manage both conditions. It is evident that in the present study, around 45% of those with cardiac comorbidity suffered from diabetes distress, with a statistically significant association between them. Similarly, a study conducted by Fisher et al. found that factors such as the increased emotional burden of managing both chronic conditions and the complexity of treatment can lead to chronic stress, resulting in distress among patients [2]. In a study conducted by Huang et al., it was found that all-cause mortality due to cardiovascular disease was higher among diabetic individuals with distress compared with diabetic individuals without distress and those without T2DM. If confirmed by further research, this causal relationship underscores the need to alleviate psychological distress among patients with T2DM to reduce the cardiovascular mortality and morbidity associated with the condition [31].

There was a statistically significant relationship found between diabetes distress and physical inactivity in the present study. There always exists a bidirectional relationship between physical inactivity and diabetes distress. Physical inactivity can lead to worsening of T2DM, leading to an increase in the glycemic status, which could lead to poor prognosis, and ultimately, failure in the management of T2DM. This lowers motivation, and fear of complications related to uncontrolled T2DM may lead to depression and anxiety. Higher diabetes distress can lead to avoidance of physical activity in the form of exercise, and can lead to negative self-talk about themselves, which may, in turn, lead to poor glycemic control [32].

Strengths and limitations

Unlike hospital-based studies, this research was conducted in an urban community setting, thus offering a more realistic estimate of the prevalence and determinants of diabetes distress in the general population. A wide range of socio-demographic, behavioral, clinical, and comorbidity-related factors were analyzed in the study, thus providing a holistic understanding of the contributors to diabetes distress. The study focuses on a relatively neglected, but highly relevant, aspect of diabetes management, especially in resource-poor settings where access to psychological support is minimal.

Since this was a cross-sectional study, no causality between risk factors and diabetic distress can be established. Most of the participant responses were based on self-reports and were thus susceptible to either social desirability or recall bias. Assessment was solely based on questionnaires and did not include formal clinical evaluation by mental health professionals, which could have provided additional diagnostic value. Participation was voluntary; individuals experiencing a higher emotional burden may have been more motivated to participate, perhaps overestimating the observed prevalence.

## Conclusions

It was found that 35.6% of the patients with T2DM suffered from diabetes distress, indicating a significant, but mostly unrecognized, health problem. It was found to be higher among females, the unemployed, and those engaged in unskilled or semi-skilled work. Inadequate spousal support led to higher levels of emotional burden. Neuropathy, foot ulcers, retinopathy, and cardiac diseases were complications that further increased distress levels. In fact, these findings suggest the interrelating consequences of clinical, psychological, and social issues in diabetes management. This condition warrants routine screening as part of the diabetes care services. Distress management should be incorporated into the National Program for Non-communicable Diseases (NP-NCD) to enable early identification and support. It is possible to develop better treatment adherence and improvement in the quality of life by strengthening community healthcare.

## Appendices

I. Socio-demography

1) Participant ID: 2) Age: 3) Gender:

4) Educational qualification:

i. Professions of honour/post-graduate ii. Graduate/diploma iii. High School iv. Middle School v. Primary School vi. Illiterate

5) Occupation:

i. Professionals/manager ii. Technicians/skilled worker iii. Clerks/shopkeepers/market sales worker/semi-skilled worker iv. Unskilled worker v. Unemployed

6) Marital status:

i. Never married ii. Married iii. Separated iv. Widowed

7) No. of family members living with him/her: ___ 8) Total family income per month: ___ 9) Religion: Hindu/Muslim/Christian/Atheist/Other

II. Diabetes history

Duration of diabetes: System of medicine: Allopathy/Homeopathy/Ayurveda/Siddha Treatment: OHA/Oha+insulin/insulin/not on medication

III. Complication history

Peripheral neuropathy: Yes/No Diabetic foot ulcer: Yes/No Diabetic retinopathy: Yes/No Diabetic nephropathy: Yes/No Cardiac complications: Yes/No

IV. Comorbidities history

Hypertension: Present/Absent Heart diseases: Present/Absent Stroke: Present/Absent

V. Self-care activities

Physical activity: Adequate/Inadequate Diabetic diet: Following/Not following Regular medication: Regular monitoring of blood glucose/follow-up as advised by the doctor: Monthly medical expenditure for diabetes:
